# Gaza midwives’ experiences in providing maternity care during COVID-19

**DOI:** 10.18332/ejm/150490

**Published:** 2022-08-04

**Authors:** Suha R. Baloushah, Nidal Abu-Hamad, Nooredine Mohammadi, Areefa S. M. Alkasseh, Motasem S. Salah

**Affiliations:** 1 Midwifery Department, Faculty of Nursing, Islamic University of Gaza, Gaza City, Palestine; 2 Department of Reproductive Health, School of Nursing and Midwifery, Tehran University of Medical Science, Tehran, Iran; 3 Independent Reproductive Health Researcher, Gaza City, Palestine; 4 Nursing Care Research Center, School of Nursing and Midwifery, Iran University of Medical Science, Tehran, Iran; 5 Nursing and Health Sciences Department, University College of Applied Sciences, Gaza City, Palestine; 6 Faculty of Public Health, Al-Quds University, Jerusalem, Palestine

**Keywords:** Palestine, COVID-19, phenomenology, midwives, maternity care

## Abstract

**INTRODUCTION:**

The Gaza Strip is densely populated. The COVID-19 pandemic has had a detrimental impact on global healthcare systems, and midwifery practices have transformed in maternity care settings. Our research aimed at understanding the Palestinian midwives’ experiences in providing maternity care in Gaza during the COVID-19 pandemic at Gaza European Hospital which was the only hospital providing care for people diagnosed with COVID-19.

**METHODS:**

To understand the phenomenon of interest, descriptive phenomenology was used. A purposive sample of eight midwives from the European Gaza Hospital was chosen. Between December 2020 and January 2021, semi-structured interviews were used in the data collection procedure. The collected data were analyzed using the Colaizzi approach.

**RESULTS:**

The investigation resulted in three main themes: emotionally overwhelmed, work environment challenges, and interpersonal relationship development.

**CONCLUSIONS:**

Midwives shared both positive and negative experiences from their time working in the European Gaza Hospital during the COVID-19 pandemic. They were confronted with negative emotions such as fear, stress, and anxiety, as well as environmental challenges. Despite this, they created a new interpersonal bond that was positively reflected. To sustain their psychological well-being, COVID-19 care workers require psychological support at work. A strong need exists for equipping the Maternity Department with the essential equipment and supplies to reduce the working environment load, as well as giving the necessary training to staff to be qualified enough to provide such crucial care.

## INTRODUCTION

The new coronavirus (SARS-CoV-2) produced COVID-19 infections, which first appeared in late 2019 in Wuhan, China, and later declared a global pandemic by the World Health Organization in March 2020^1^. Low- and middle-income countries, in particular, are facing numerous challenges in dealing with the COVID-19 disastrous implications. Job and income loss, fear, stigma, and future uncertainty are just a few of the issues that people face^2,3^. Furthermore, the ability of the existing under-resourced health systems to cope with the COVID-19 increased load is crucial^4^. During the early stages of the COVID-19 pandemic in Hubei, more than 3000 medical workers were infected, with 40% being hospital medical staff, due to a lack of awareness of the virus transmission mechanisms, prevention, and control methods^5^.

The Gaza Strip is a densely populated area, with an estimated population of 2.11 million in 2021^6^. Around 178452 people had been infected with COVID-19 by the middle of October 2021. The first two coronavirus-positive cases in Gaza were discovered in late March 2020, at the border of the Gaza Strip with Egypt^7^. In August 2020, the first communal case was reported. Since February 2021, the number of cases has increased^8^. It is reported that around 441882 people have been infected with COVID-19 as of mid of October^9^. COVID-19 had the impact of increasing the workload of maternity services by midwives who were providing care during the pandemic. Health officials will benefit from this information as they decide how to sustain the healthcare system during the pandemic and support the midwifery workforce^10^. According to the literature, there is a pressing need to fill the gaps in information about Gaza midwives' experiences during COVID-19 at the European Gaza Hospital. In addition to pregnant women with COVID-19, the Palestinian Ministry of Health designated the European Gaza Hospital for all COVID-19 patient treatment despite challenges due to lack of essential supplies and equipment^11^. To the best of our knowledge, there is currently no research that has produced such information as the current study aims to investigate. The goal of this study is to learn more about the midwives’ experiences in providing maternity care at Gaza European Hospital during COVID-19.

## METHODS

### Design

In our research, we used a descriptive phenomenological approach. It was used to arrive at fundamental meanings by delving deeply into reality to maintain the phenomenon’s objectivity^12-14^. To examine the Gaza midwives’ experiences in providing maternity care in maternity wards at European Gaza Hospital during the COVID-19 pandemic, a descriptive phenomenology was used.

### Study sample and sampling strategy

Midwives who worked in the maternity wards at European Gaza Hospital during the COVID-19 epidemic and provided maternity care to women with COVID-19 were included in the current study. The study participants were chosen using a purposive sample strategy based on the inclusion criteria. Midwives who had firsthand experience with the topic under investigation and had an interest in participating in the study were recruited. In addition, in this investigation, a snowball sampling strategy was used. The research comprised a total of eight midwives (Table 1).

**Table 1:** Characteristics of the study participants (N=8)

*Study participants*	*Age (years)*	*Experience (years)*	*Academic degree*	*Interview duration (min)*
P1	25	2	Master’s	50
P2	27	4	Bachelor’s	45
P3	26	3	Bachelor’s	40
P4	32	10	Diploma	30
P5	26	5	Bachelor’s	55
P6	24	2	Bachelor’s	50
P7	27	4	Master’s	45
P8	25	2	Bachelor’s	30
Mean±SD	26.5±2.4	4±2.6		

### Data collection

To obtain data, semi-structured interviews were conducted. The interviews were performed and recorded through Zoom by the lead researcher between December 2020 and January 2021 due to COVID-19 restrictions. After introducing and discussing the study’s goal, study participants were asked to share their experiences in providing maternity care during the pandemic. One of the open-ended guiding questions utilized by the lead researcher was: ‘Can you tell me about how you give maternity care to attended women during the COVID-19 pandemic as a midwife?’. As the interviews progressed, more precise and descriptive questions were asked such as: ‘Tell me more about your feelings’.

The interviews lasted from 30 to 50 minutes and were transcribed verbatim immediately after they were completed. The data collection proceeded until the data were saturated and no new meaning units emerged from the transcripts of the participants.

### Data analysis

The data were analyzed using the descriptive Colaizzi approach^15^. The following are the seven steps in this method: 1) collecting the participants’ descriptions, 2) comprehending the depth of the meanings, 3) extracting the important sentences, 4) conceptualizing important themes, 5) categorizing the concepts and topics, 6) constructing comprehensive descriptions of the issues examined, and 7) validating the data (Table 2).

**Table 2:** The stages of data analysis using the descriptive Colaizzi method

*Stages*	*Researcher activity*
Collecting the participants’ descriptions	Zoom meeting and recording of the interviews
Understanding the depth of the meanings	Transcription and re-reading of the interview transcript to gain in-depth understanding of the phenomena
Extracting the important sentences	Reviewing the midwife explanation and extracting the important sentence
Conceptualizing important themes	Extracting the important explanation and giving them meaning with specific concept
Categorizing the concepts and topics	The obtained concept placed in specific categories
Constructing comprehensive descriptions of the issues examined	Clear and unambiguous expression of the phenomena under study
Validating the data	Lincoln and Guba’s criteria used

### Trustworthiness

Lincoln and Guba’s four criteria were used: credibility, confirmability, dependability, and transferability to ensure rigor in the current study^16^. A member check was used to establish credibility. The study participants were asked to give their feedback about analytic categories, interpretation, and conclusion. Eight midwives with experience in caring for COVID-19 mothers were chosen to provide comprehensive data suitable for transferability. To attain dependability and conformability, an audit trail was followed. The research step described from the start to the development and reporting of the finding. The record of the research path was kept throughout the study.

### Ethical considerations

The Palestinian Health Research Council and the Ministry of Health granted ethical approval for this study. Permission was obtained verbally and signed as informed consent from participants who agreed to participate in this study. Before the consent form was signed, oral and written information was provided. It stated that they have the option to exit the interview if they do not wish to continue.

## RESULTS

The current study engaged the participation of eight people. The participants were on average 26.5 years old, with the majority holding a Bachelor’s degree or higher. The study participants had an average of four years of work experience providing maternity care, details characteristics are presented in Table 1. Three primary themes and six subthemes emerged from the participants’ experiences in providing maternal care during the COVID-19 pandemic (Table 3).

**Table 3:** Themes and subthemes emerged from data analysis

*Themes*	*Subthemes*
Emotionally overwhelmed	Anxiety and stress
	Fear from outcome
Care provision context	Lack of preparedness
	Turmoil
Interpersonal relationship growth	Coworker relationship development
	Midwife–mother relationship development

### Emotionally overwhelmed

The participants talked about their emotional responses to caring for pregnant ladies and women in childbirth. The participants also described their feelings of worry and tension when delivering care, as well as their feelings of fear.

*Overwhelmed stress and anxiety*

The participants expressed their feelings of tension and worry when providing treatment to women in labor or pregnant women who stayed in the department for medical management, as well as from the maternal outcome due to respiratory distress syndrome:

*‘At the first shift, while I'm taking care of a pregnant mother, I felt anxious.’* (P6)

Another participant also declared that the whole situation made her feel stressed. She pointed out:

*‘The whole situation was stressful; it was not easy for me. Everything was new.’* (P4)

*Overwhelmed by fear*

Participants expressed their anxiety of being in a new situation with unknown outcomes, as well as their worry of becoming infected and losing their lives:

*‘At first, I was thinking about surrendering since I was afraid that I would catch the infection as well. I don't want to lose my life, and I certainly don't want to die.’* (P6)

They dreaded social rejection from relatives and neighbors when they learned that the participants worked in a European hospital specialized in caring for COVID-19 patients:

*‘I was afraid of being socially isolated when I return home because my relatives and neighbors might stop interacting with us because of working in a place that had COVID-19 cases.’* (P3)

*‘I was afraid of many things, including staying away from my family for a long time, I was also afraid of getting a COVID-19 infection as I worked in the hospital.’* (P4)

### Care provision context

The participants described their workplace turbulence and lack of preparedness requiring support and equipment in care provision for expectant mothers while in the hospital or during labor.

*Lack of preparedness*

A participant claimed that the department lacked documents on obstetrics and gynecology:

*‘At first, there was a paucity of maternity-specific documents … We usually request documents from the supervisor.’* (P6)

*‘I used to note down mother's progress on medical files, but due to a lack of files, I missed a lot of information.’* (P5)

*‘When I monitored the fetal heart rate, there was a shortage of CTG paper to document the fetal heart rate.’* (P3)

*‘One time we required a vacuum since there was a bradycardia, but it was not available in the department.’* (P8)

*Turmoil*

Participants reported their feelings of being unqualified for such work due to a lack of experience and the stress of working under such conditions:

*‘At the beginning of working, maternity cases were not there is a separate section, they were present among non-pregnant cases, I assisted in taking care of non-pregnant cases, the workload was too heavy.’* (P1)

They were overburdened by responsibilities because they were in charge of coordinating everything linked to care delivery:

*‘There was too much work, I'm accountable for everything in the department.’* (P7)

Midwives felt unqualified due to a lack of experience in situations requiring particular skills and education:

*‘I did not have enough experience in infection prevention measures, and I did not take the infection control training, I didn't think I was qualified’*. (P2)

Participants found it difficult to provide adequate care due to a lack of training and expertise:

*‘At first, it was challenging because everything was new to me.’ Because I didn't get adequate training, it was a new experience for me.’* (P1)

### Interpersonal relationship development

Participants formed new relationships with other medical professionals from various hospitals while working at the European Hospital. During their extended stay in the hospital, they also developed positive relationships with the women. After the women were discharged, they were able to use them as personal midwives because of their friendship.

*Coworker relationship development*

Participants expressed their delight at developing new professional contacts:

*‘I was thrilled at the end of my time at the European Hospital since I met new doctors and other colleagues from different hospitals around the Gaza Strip.’* (P8)

*‘I've formed new relationships with new colleagues from other hospitals and this has given me a new experience in a different field of practice.’* (P5)

*Midwife–mother relationship development*

During hospitalization, relationships developed between participants and mothers, with midwives demonstrating empathy for the mothers during caregiving, despite the midwives serving as personal midwives after discharge. The women continued to contact, seek advice and consultation from the midwives:

*‘I was so happy when some moms were continuing contacting me even after discharge. They made me feel like a useful person.’* (P2)

*‘I used to help all of the mothers as possible as I can, I tried to provide them with anything they need when their family cannot access them. We became a family during staying in the hospital’*. (P3)

### They continued to communicate with them and seek their opinion and consultation:

*‘I was thrilled when some moms continued to contact me even after they were discharged.’* (P2)

*‘They made me feel like a valuable member of the team, I used to help all of the mothers as much as I could, I tried to provide them with anything they needed when their relatives couldn't access them.’* (P3)

## DISCUSSION

There is a scarcity of qualitative research on midwives’ experiences in caring for women during pregnancy or childbirth during COVID-19. This study looked at the experiences of Palestinian midwives living in Gaza while working at the European Gaza Hospital providing maternal health services during the COVID-19 pandemic. The key themes that arose from the midwives’ descriptions of their experience were outlined.

Midwives in the Gaza Strip are driven by a strong professional conscience to provide the best possible care during childbirth, despite a difficult work environment, low professional status and poverty making them psychologically unsuitable to take on such a stressful duty. Fear and anxiety from a new experience, as well as worry of unexpected results during care provision, infection, and death, as well as concern of maternal implications, social consequences of working in quarantine hospital, were all expressed by midwives in the current study. Previous studies have also shown these emotional manifestations^17-19^.

The participants were quite concerned about the mothers’ health, which was also revealed in a prior study^20^. According to several studies, healthcare personnel who worked on the front lines with COVID-19 patients felt similar emotions^21^. According to a study, working in a stressful atmosphere harms the mental and emotional health of healthcare providers^22,23^. Midwives in this study found difficulties when providing care because they did not feel qualified and lacked expertise. Previous studies have shown that these emotional manifestations exist^18,22^.

Workplace issues impose strain on caregivers; these challenges arose from a lack of preparedness, such as the lack of necessary supplies and equipment for maternal services. This outcome is consistent with past research findings^4,24,25^. Furthermore, emotional anguish, work obstacles, and a lack of needed supplies and equipment will have an impact on the quality of care and put patient safety in jeopardy^26,27^.

Resilience amid challenges was experienced by midwives who participated in the current study; they discussed their support for one another and the coping solutions they used, which were stated in previous research^27,28^. Midwives in this study claimed that spending a long time in the hospital with mothers, resulted in a professional and personal relationship that lasted after they were discharged. The midwives felt that establishing a new positive relationship with other practitioners had a favorable impact on their experience. The improvement in teamwork boosted the favorable work environment, which has been linked to managing professional resilience in the face of adversity in earlier studies^10,29,30^. The study’s findings revealed that caring for COVID-19 patients is difficult and has significant emotional and professional implications^31,32^.

### Strengths and limitations

Our study’s strength was that it was the first of its kind to be conducted among midwives in Gaza. The study’s biggest constraint was employing an alternative strategy for conducting interviews using the Zoom application to bypass the study participant’s access barrier due to the COVID-19 restriction.

## CONCLUSIONS

Fear, worry and anxiety were expressed by midwives working at the European Gaza Hospital. They faced hurdles in the care environment and formed interpersonal bonds. Midwives require psychological support in the professional context of COVID-19 care to maintain their mental health. The midwives highlighted the need for preparing the Maternity Department by procuring the essential equipment and supplies to reduce the workload in the working environment, as well as giving the necessary training to be competent to provide such critical care. Based on the findings, we propose conducting additional studies on the coping methods used by midwives in healthcare settings to help them cope with these challenging circumstances.

## Data Availability

The data supporting this research are available from the authors on reasonable request.
